# Dietary intake and adherence to the Mediterranean diet in semi-professional female soccer players: a cross-sectional study

**DOI:** 10.3389/fnut.2024.1378365

**Published:** 2024-04-19

**Authors:** Alessandro Modena, Maria Cristina Casiraghi, Daniela Erba

**Affiliations:** Department of Food, Environmental and Nutritional Sciences (DeFENS), Università degli Studi di Milano, Milan, Italy

**Keywords:** energy intake, nutrient intake, Mediterranean diet adherence, soccer, female players

## Abstract

**Background:**

Adequate energy and nutrient intakes in athletes contribute to optimal performance and recovery, decrease the risk of injury, and help preserve athletes’ health. The Mediterranean diet (MD) is considered suitable for covering the nutritional needs of athletes, while contributing to improve eating habits. The aim of the present study was to investigate the energy and nutrient intakes of semi-professional female soccer players and their adherence to the MD, during the competitive season.

**Methods:**

A cross-sectional observational study was conducted on twenty-three female soccer players, who were invited to fill in a 3-day food diary twice, one month apart, to assess their energy and nutrient intakes and a validated questionnaire (MEDI-LITE) to evaluate their adherence to MD. Exercise energy expenditure during three training and match-play days was monitored by GPS.

**Results:**

On average, the participants consumed 1,981 kcal/day, with 44% of their energy deriving from carbohydrates, 21% from protein, and 34% from fat; the mean MD adherence score was 10.1 ± 1.8, corresponding to a good MD adherence. A substantial percentage of athletes were at risk of insufficient nutrient intakes for vitamin D (100%), iodine (87%), potassium (87%), vitamin E (39%), iron and zinc (17 and 30%, respectively).

**Conclusion:**

The evaluation of the dietary intake in female soccer players showed energy deficiency in relation to training level, mainly due to the insufficient intake of carbohydrates, and micronutrient deficiencies. The technical staff should promote adequate consumption of starchy foods in female athletes and emphasize the importance of a proper distribution of energy intake among different eating occasions, including snacks. Periodic monitoring of the nutritional status of micronutrients (vitamin D and some minerals) could help reduce deficiency risk. Over all, nutrition education to improve eating habits of athletes seems worthy of attention, in order to preserve athletes’ health and performance.

## 1 Introduction

Soccer is one of the most popular sports in the world, especially the men’s competitions, while the attention given to women’s tournaments has been increasing in recent years. Female soccer is considered professional in Italy from 2022, and its popularity among teenagers and the number of teams and players have been steadily growing. In Italy, the number of female footballers registered with the FIGC (Italian Football Federation) has doubled in the past 5 years (1).

Soccer is considered an intermittent aerobic sport, characterized by high-intensity activities (sprinting, directional changes, and tackles/duels) separated by low- to moderate-intensity actions and short/insufficient recovery intervals (2). The total energy expenditure (EE) is estimated to be between 1,200 and 1,500 kcal/day during a match in men, whereas in women it is approximately 30% lower (3). In female, average activity EE ranged between 418 ± 140 kcal/day (881 ± 473 in the match days) (4) to 1,058 ± 352 kcal/day (5). The energy demand mainly differs according to body weight (bw)/composition of athletes and playing position (6): for instance, higher EE was assessed in midfielders (1,318 ± 195 kcal/day) than in defenders and attacks (964 ± 436 and 1,073 ± 348 kcal/day, respectively) (5). Training improves the physical performance in both male and female soccer players, resulting in similar differences compared with their sedentary counterparts (7).

Adequate nutrient intakes in athletes contribute to optimal performance and recovery, decrease the risk of injury, and help preserve athletes’ health. At the professional level, it is common to find nutrition experts collaborating with technical teams; these figures are usually absent in semi-professional or lower sport divisions, where coaches or, in the best cases, occasional external nutritionists, are responsible for nutrition counselling. Furthermore, due to their high motivation and, on average, low nutritional knowledge, athletes are more receptive than ordinary people to nutritional messages even when conveyed from non-professional sources (e.g., social media), thus being more at risk for unhealthy eating behaviors. The prevalence of eating disorder appears to be high in elite athletes, especially in females competing in aesthetic or weight-class disciplines, but eating disorder has also been detected in team sports (8).

Inadequate nutritional intake has often been reported in both male and female soccer players. In the review by Garcia-Roves et al., an inadequate energy intake (EI) was found in soccer players, regardless gender, due to insufficient carbohydrates intake (9). Over the last 10 years, several studies have investigated the dietary intake of these athletes, mostly on male players, and nutritional deficiencies have often been confirmed. The review of Danielik et al. (10), by analyzing studies on dietary intake of male soccer players in pre-season period and during the season, found inadequate energy and carbohydrates intakes not in line with sport-nutrition recommendations, while the majority of athletes had adequate protein and fat intakes. A recent study on Spanish male soccer players from different categories (11) highlighted that Super League players had higher intakes of energy, protein (g/kg bw) and carbohydrates (g/kg bw) and lower fat intake (as percentage on total EI) compared to the semi-professional players, who did not meet UEFA recommendations for energy and carbohydrates, but exceeded that for fat intake (34% EI) (12). Finally, even though nutrition is normally considered an important tool for optimizing performance, the eating habits and nutritional knowledge in athletes and coaches are sometimes unsatisfactory, exacerbating the risk of malnutrition (13).

The Mediterranean diet (MD) is one of the most studied dietary patterns, and its beneficial effects on health and disease prevention have been extensively investigated in comparison with other popular diets (14, 15). This dietary pattern is based on high consumption of plant-based foods such as fruit, vegetables, and cereals, with moderate consumption of animal foods. Recently, an observational study performed on Spanish university students demonstrated that subjects with a high MD adherence, in addition to having significantly higher intake of protein and lower intake of lipids, had the highest cardiorespiratory and muscular fitness levels (16). Notwithstanding, other studies have raised concerns about the adequacy of MD to meet the protein needs of athletes (17).

Based on these premises, the present study aimed to investigate dietary energy and nutrient intakes (macro- and micro-nutrients), the daily distribution of energy, protein, branched chain amino acids (BCAA) and leucine intakes and adherence to MD, in semi-professional female soccer players during the competitive season.

## 2 Materials and methods

### 2.1 Experimental design

A cross-sectional observational study was performed on female athletes from the first division of the Florentia San Gimignano (Tuscany, Italy) soccer team, who were invited to participate in the study. The recruitment was carried out through coaches, after explaining the purpose of the study, who supported participants’ compliance. In the month before the start of the study, the athletes were asked to fill out a short personal data sheet (weight, height, particular eating habits, food intolerances or allergies). Exclusion criteria were: the presence of food allergies/intolerances and adherence to a vegan or a more restrictive diets. The study was performed between March and May 2021. Dietary intake and MD adherence were recorded twice, one month apart, and exercise EE in training and match-play days was monitored 3 times for each condition. Resting metabolic rate (RMR) of athletes was estimated by means of a prediction equation validated by Watson et al. (18) in similar female athletes; the equation was then applied to estimate energy needs (physical activity level -PAL- = 1.75).

A total of 23 semi-professional female soccer players, committed to Italy’s top women’s tournament (Serie A), were recruited. Physical characteristics of the participants, such as age, weight, height, were self-reported by the athletes. All participants were over 18 years of age, were informed about the nature of the study and signed written informed consent. The Ethics Committee of the Università degli Studi di Milano approved the study protocol (protocol number 62/21).

### 2.2 Dietary record

Athletes filled in the 3-day food diary twice, between March and May 2021, within the competitive season. The 3-day diary was given to the athletes in paper format; it was structured into the various daily eating occasions (breakfast, morning snack, lunch, afternoon snack, dinner and after dinner snack) for which time and place of food consumption, their description and quantity are required (19). The 3-day food record consisted of 2 workdays and one rest day for both collections. The participants received verbal and written instructions for completing the food record, including information for quantifying the serving size and household measures (teaspoon or tablespoon etc.), and were invited to fill in the food diary during meal time. Over the two months of the study, a nutritionist was present in the field to support the volunteers with the compilation of the food diary and assess it (for instance, reminding them about the meal occasions for food consumption, the brand names of food, cooking method, and any other useful information). If necessary, the expert used a Photographic Food Atlas for quantification purposes (20). Athletes were provided with a model of a corrected dietary record as an example of the detailed description required.

### 2.3 Dietary analysis

A single researcher undertook the dietary analysis using the Metadieta^®^ software (Me.te.da srl, Roma, Italy) to assess the total EI, macro- and micro-nutrients, and daily distribution of energy, protein, and BCAA intakes. This software is based on the BDA Food composition database, that provides the composition for 978 Italian food items, hugely applied in nutritional studies in Italy (21). The athletes did not consume any nutrient supplementation, only with the exception of someone who sporadically drank electrolyte sports drinks.

### 2.4 MD adherence

All the 23 athletes completed a validated MD adherence questionnaire (MEDI-LITE) (22, 23) at both times. The MEDI-LITE is a literature-based questionnaire built on the quantities of food consumption consistent to adherence to MD in the cohort studies considered by Sofi et al. (23). From these data, the Authors identified three categories of consumption for each food group: fruit, vegetables, legumes, cereals, fish, meat and meat products, dairy products, alcohol and olive oil. Consumption adhering to the MD was scored 2, while 1 or 0 points were assigned when consumption became progressively misaligned with the Mediterranean eating pattern.

### 2.5 Exercise energy expenditure during training and match-play

During 3 days of training in the field and 3 days of match-play, the exercise EE of players was monitored by GPS (K-Sport Universal, Italy), to highlight differences due to playing position, excluding goalkeepers (24).

### 2.6 Statistical analysis

Statistical analysis was performed by STATISTICA software (release 6.0 StatSoft Europe, Hamburg, Germany). The normal distribution of data was assessed by Shapiro–Wilk test (*p* < 0.05). The differences of macronutrient energy contribution in high and low MD adherent groups were analyzed by *t*-test (*p* < 0.05). The effect of playing position on distance and EE in training and match days was analyzed by analysis of variance (ANOVA). *Post hoc* analysis of difference was assessed by least significant differences (LSD) test, with *p* < 0.05 as level of statistical significance. Data are expressed as mean ± sd (standard deviation).

## 3 Results

### 3.1 Participant characteristics and activity

A total of 23 female athletes were enrolled in the study. Table 1 reports the physical characteristics of the volunteers, such as age, weight, height, and BMI, self-reported by the athletes, as average of all players and in relation to playing position. All the athletes were of normal weight with a BMI between 19.4–25.2 kg/m^2^, the highest figure was for a goalkeeper. The weekly physical activity program for athletes included 5 days of soccer training on the field, 1 day of rest, and 1 match day. Furthermore, twice a week, athletes followed a 2-h gym training session. The soccer team comprised three goalkeepers, three defenders, four fullbacks, eight midfields, and five forwards.

**Table 1:** Physical characteristics of female soccer players (*n* = 23).

	All (*n* = 23)	Goalkeepers (*n* = 3)	Defenders (*n* = 3)	Fullbacks (*n* = 4)	Midfields (*n* = 8)	Forwards (*n* = 5)
Age (y)	23.8 ± 3.7	24.3 ± 3.5	27.7 ± 4.0	23.3 ± 3.9	23.0 ± 3.7	23.2 ± 3.7
Height (cm)	167 ± 4	169 ± 4	171 ± 7	165 ± 1	166 ± 3	165 ± 4
Weight (kg)	60.6 ± 5.1	69.0 ± 5.3	62.5 ± 6.5	58.3 ± 1.3	58.3 ± 3.7	59.9 ± 3.6
BMI (kg/m^2^)	21.8 ± 1.3	24.0 ± 1.1	21.4 ± 1.8	21.4 ± 0.7	21.2 ± 0.8	22.1 ± 0.9

Values are expressed as mean ± sd (standard deviation); BMI, body mass index.

### 3.2 Energy and nutrient intakes

Mean daily total energy (kcal/day), macronutrient (g/day, g/kg bw, % EI), dietary fiber (g/day) and micronutrient (mg or μg/day) intakes are shown in Tables 2, 3.

**Table 2:** Daily intakes of total energy and nutrients in female soccer players (*n* = 23).

	Mean ± SD	Recommendation
Energy		
kcal/day	1,981 ± 395	
kcal/kg bw/day	32.9 ± 7.1	47–60 kcal/kg bw/day (25)
Carbohydrates		
g/day	226 ± 54	
g/kg bw/day	3.7 ± 1.0	6–10 g/kg bw/day (26)
% total kcal	44 ± 4	45–65% EI (27)
Free sugar		
g/day	77 ± 19	
% total kcal	15 ± 3	<15% EI (27)
Protein		
g/day	99 ± 14	
g/kg bw/day	1.6 ± 0.3	1.4–2.0 g/kg bw/day (28)
Fat		
g/day	74 ± 20	
% total kcal	34 ± 5	20–35% EI (27)
SFA% total kcal	10 ± 3	<10% EI (27)
MUFA % total kcal	13.5 ± 2	
PUFA % total kcal	5 ± 2	5–10% EI (27)
Cholesterol (mg/day)	267 ± 111	<300 mg (27)
Dietary Fiber (g/1000 kcal/day)	28 ± 9	12.6–16.7 g/1000 kcal/day (27)

Values are expressed as mean ± sd (standard deviation); bw, body weight; EI, energy intake; SFA, saturated fatty acids; MUFA, monounsaturated fatty acids; PUFA, polyunsaturated fatty acids.

**Table 3:** Daily micronutrient intakes in female soccer players (*n* = 23) and number (percentage) of athletes at risk of insufficient nutrient intakes.

Nutrients	Mean ± sd	DRV (27)	*n* (%) of athletes at risk of insufficient intake
Iron (mg)	11.7 ± 2.8	18^a^	4 (17%)^c^
Calcium (mg)	954 ± 213	1,000^a^	6 (26%)^c^
Sodium (mg)	1766 ± 556	1,500^b^	6 (26%)^d^
Potassium (mg)	3,167 ± 814	3,900^b^	20 (87%)^d^
Zinc (mg)	9.0 ± 2.3	9^a^	7 (30%)^c^
Iodine (μg)	86 ± 55	150^b^	20 (87%)^d^
Vitamin A (RE μg)	1,384 ± 574	600^a^	0 (0%)^c^
Vitamin E (mg)	13.1 ± 4.1	12^b^	9 (39%)^d^
Vitamin D (μg)	3.1 ± 2.1	15^a^	23 (100%)^c^
Ascorbic acid (mg)	175 ± 77	85^a^	2 (9%)^c^
Thiamin (mg)	1.2 ± 0.3	1.1^a^	4 (17%)^c^
Riboflavin (mg)	1.6 ± 0.4	1.3^a^	0 (0%)^c^
Folate (μg)	371 ± 130	400^a^	7 (30%)^c^

Values are expressed as mean ± sd (standard deviation); DRV, dietary reference values; RE, retinol equivalent.

a PRI population reference intake (27).

b AI adequate intake (27).

c Number and percentage of athletes with intake lower than average requirement (AR) (27).

d Number and percentage of athletes with intake lower than adequate intake (AI) (27).

Participants consumed 1,981 ± 395 kcal/day (mean ± sd) with 44% of energy coming from carbohydrates, 21% from protein, and 34% from fat. Most of the EI occurred with dinner and after dinner snacks: 22% of players consumed more than 40% of their daily EI at dinner. Similarly, protein and BCAA intakes were unequally distributed across meals (Table 4).

**Table 4:** Daily distribution of total energy, protein, BCAA and leucine intakes, in female soccer players (*n* = 23).

	Energy (% of daily intake/meal)	Protein (g/meal)	BCAA (g/meal)	LEU (g/meal)
Breakfast	18 ± 4	15.1 ± 5.8	1.4 ± 0.7	0.6 ± 0.3
Morning snack	1 ± 1	0.4 ± 0.6	0.0 ± 0.0	0.0 ± 0.0
Lunch	33 ± 5	32.1 ± 10.5	3.7 ± 1.9	1.6 ± 0.9
Afternoon snack	13 ± 5	10.5 ± 4.3	0.6 ± 0.4	0.2 ± 0.1
Dinner and after dinner	35 ± 6	41.2 ± 11.0	5.5 ± 2.4	2.4 ± 0.8

Values are expressed as mean ± sd (standard deviation); BCAA, branched chain amino acids; LEU, leucine.

The average athletes’ carbohydrate intake was lower than the reference values (3.6 g/kg bw/day vs. a range of 6–10 g/kg bw/day recommended for a mod-high intensity exercise program) (26). Actually, 91% of athletes did not achieve the recommended carbohydrate intake even for moderate intensity (5–7 g/kg bw/day) (26). The intakes were consistent with the dietary guidelines for protein, cholesterol, fat and dietary fiber. Regarding micronutrients, athletes displayed insufficient intakes of iron, iodine, potassium, folate and vitamin D with respect to the Dietary Reference Value for the Italian population (LARN) (27) (Table 3). Notably, all athletes were at risk for insufficient intake of vitamin D, 87% at risk for potassium and iodine, 39% for vitamin E, 30% for folate and zinc and 17% for iron.

### 3.3 Energy expenditure

Table 5 reports the mean distance covered and the mean exercise EE of the players for three training days and three match days, in relation to their playing position. Despite this device generally underestimates EE in intermittent exercise, it provides useful information for estimating energy availability (EA) (24). The recorded data suggest that the playing position does not affect the distance covered and EE during training (*F* = 0.0117 *p* = 0.9982 and *F* = 0.0439 *p* = 0.9869, respectively), while defenders showed higher intensity of physical activity than the midfield players during the match (for distance *p* = 0.0369 and for EE *p* = 0.0423, between these two roles).

**Table 5:** Distance covered (m) and mean energy expenditure (kcal/kg bw) of female soccer players in relation to playing position, estimated by GPS, as the mean of three training days (T) and three match days (M).

Playing position	Distance (m)		EE (kcal/kg bw)	
	T	M	T	M
Defender (*n* = 3)	6,774 ± 2,190^a^	8,022 ± 411^a^	8.6 ± 2.6^a^	10.4 ± 0.6^a^
Fullback (*n* = 4)	6,975 ± 2,152^a^	6,915 ± 1,408^ab^	8.9 ± 2.6^a^	8.9 ± 1.8^ab^
Midfield (*n* = 8)	6,665 ± 2,014^a^	6,204 ± 878^b^	8.1 ± 3.0^a^	8.1 ± 1.2^b^
Forward (*n* = 5)	6,740 ± 2,137^a^	6,519 ± 500^ab^	8.7 ± 2.5^a^	8.6 ± 0.6^ab^

Values are expressed as mean ± sd (standard deviation); EE, energy expenditure in field; T, Training day; M, match day; bw, body weight; data in the same column not sharing common letter are significantly different (*p* < 0.05). Comparisons between T and M within the same role are not significant for distance and EE.

### 3.4 Adherence to Mediterranean diet

The mean MD adherence score was 10.1 ± 1.8 (the frequencies of food group consumption are reported in Supplementary Table S1). According to the MEDI-LITE questionnaire validation study (22), a score above 8.5 discriminates adherence to the MD. In our soccer players, 74% of athletes (*n* = 17) resulted adherent to this dietary pattern, but only 13% of the athletes had a consumption frequencies of fruit, 8.7% of legumes and 13% of fish, consistent with a Mediterranean dietary pattern (Supplementary Table S1). Non-adherent athletes mainly reported consumption of fruits and vegetables less than 1 serving per day and legumes less than 1 serving a week. Although the limited number of subjects does not allow conclusions to be drawn, the comparison between high and low MD adherence diet suggests that the former provided adequate intakes of dietary fiber (30.8 ± 8.6 g/day vs. 21.4 ± 7.2 g/day, respectively, *p* < 0.05) and lower percentage of energy from saturated fatty acids (SFA) (9.5 ± 2.3 vs. 12.5 ± 2.1% EI, respectively, *p* < 0.05) than the latter, while intakes of protein (21.4 ± 5.1 vs. 21.4 ± 2.7% EI, high vs. low MD adherence), fat (33.6 ± 4.8 vs. 36.6 ± 3.8% EI) and carbohydrate (45.0 ± 3.3 vs. 42.0 ± 3.8% EI) were comparable.

## 4 Discussion

The primary purpose of this study was to assess the nutritional quality of semi-professional female soccer players’ diets and, secondly, to evaluate the MD adherence of their diet.

Data obtained from dietary recall (3-day diary repeated twice) suggest insufficient EI. We did not measure the athletes’ resting metabolic rate (RMR); thus, a prediction equation was applied to assess the adequacy of EI. Some studies in the literature analyzed the reliability of different RMR prediction equations, drawing conflicting conclusions (29, 30). In 2019, Watson et al. (18) validated a new equation in American collegiate female athletes whose age and anthropometric measures resemble our volunteers (31); the equation was then applied to estimate RMR and energy needs (PAL = 1.75). Comparing these estimated energy needs with the mean EI from dietary recall, 22 out of 23 athletes displayed energy deficiency (25% less energy). The mean relative EI of 33 ± 7 kcal/kg bw/day fell below the recommendation of 47–60 kcal/kg bw/day for female soccer players (25), in accordance with other studies (3, 25, 32–35). Two athletes (9%) did not meet 30 kcal/kg fat free mass (FFM)/day limit (assuming 24% of fat mass) (3) indicated by the American College of Sport Medicine (ACSM) as being associated with impaired physiological function (26). Even considering that women are more prone to underreport dietary intake than men (36), this inadequate EI could impair physical performance, recovery, overall nutrients intake, and health (4). Worthy of note, EI were focused on the main meals, especially dinner and after dinner snacking, which are expected to negatively affect athletes’ sleeping.

The mean intake of carbohydrates, expressed as relative to body weight, did not meet the recommended 5–7 g/kg bw/day for moderate physical activity (26). Low consumption of complex carbohydrates explains this result, considering that free sugar intake reached the upper limit of the suggested dietary target (27). Low carbohydrate intake jeopardizes the maintenance and restoration of glycogen stores, resulting in the early onset of muscle fatigue and reduced performance (37). Female athletes are more susceptible to impaired carbohydrate intake due to generally lower EI compared to men (38). According to our results, several studies reported carbohydrate intakes lower than 5 g/kg bw/day in female players (33, 35, 39, 40). To meet the lower limit of the recommended carbohydrate intake for moderate activity, female soccer players should increase EI by consuming food rich in complex carbohydrates (such as starchy food).

Otherwise, the widespread knowledge of the role of proteins in athletes’ performance during exercise could explain the adequate intake of protein found in this study. For this macronutrient, our result of 1.6 ± 0.3 g/kg bw/day was higher than literature data of 1.2–1.4 g/kg bw/day (9), but consistent with the recommended protein intake in physically active people (1.4–2.0 g/kg bw/day) (28). In addition to quantity, food frequency consumption data extrapolated from the MEDI-LITE showed that 70% of participants consumed 1–1.5 servings per day of meat and meat products, and 4% more than 1.5 servings per day. The dietary record further showed high egg consumption, as confirmed by cholesterol intake which exceeded the suggested dietary target (< 300 mg/day) (27) in 39% of the athletes. Data from MEDI-LITE showed that 70% of athletes consumed 1–2 servings per week of legumes, although only 9% met the Italian Food Based Dietary Guideline of 3 servings per week for a 2,000 kcal/day diet (41). A total of 13% of participants consumed more than 2.5 servings per week of fish, a food source of high quality protein but also essential fatty acids. In athletes, more attention should be paid to essential amino acids (EAAs) intake, especially leucine, to optimize muscle protein synthesis and recovery. The International Society of Sports Nutrition suggests that athletes should consume high-quality dietary protein, containing adequate EAAs at each meal (every 3–4 h): specifically, 20–40 g of dietary protein, 10–12 g of EAAs, and 1–3 g of leucine per meal have been proposed (28). Our results suggest that leucine intake is concentrated at main meals contrary to recommendations for elite football players (12). In addition to total protein intake, our results showed an unbalanced distribution of protein intake among meals. Morning snacks provided insufficient amounts of protein, while the main intake came from dinner and after dinner snacks. Consistent with our findings, Kwon et al. found that dinner was the highest protein containing meal, whose intake was uneven distributed across meals, in both male and female soccer players (42).Even though the low number of subjects involved, the present results suggest that athletes adequately met total protein requirements, but food sources and timing should be better emphasized.

There are no specific recommendations for fat intake in athletes, which usually resemble those of the general population (43). However, individualization of fat intake should be appropriate in relation to training intensity or body composition goals. Excessive EI from lipids is unsuitable for athletes, as it affects overall macronutrients balance and reduces performance (44). Similarly, excessive lipid reduction may have detrimental impacts on the availability of lipophilic nutrients (i.e., Vitamin D and essential fatty acids). The soccer players involved in our study had 34% of their energy from fat, higher than the value recommended by Clark et al. (45), but not by Thomas et al. (26), and higher than values reported in other studies in professional female soccer players (4, 39). Nonetheless, this percentage of fat on total EI is comparable to that of Italian adult female population (46). Note that the average intake of saturated fatty acids is at the upper end of the Italian DRVs, while polyunsaturated fatty acids are at the lower end of the reference range (27).

The low EI in athletes’ diet was likely related to the large proportions of athletes at risk of insufficient intake of micronutrients, such as vitamins D, E and folate, and key minerals like iron, zinc and potassium. Low vitamin D intake has already been shown in female football players (33, 35) and it is common in the adult Italian female population, too (46). In this regard, it is interesting to note that football is an outdoor discipline; therefore, insufficient dietary intake of vitamin D may be counteracted by endogenous synthesis. Nevertheless, the risk of low vitamin D and Ca intakes should gain proper attention because of the negative impact on players’ skeletal systems and future bone health.

A discrepancy between EI and total EE has often been reported in studies on female soccer players. For example, in the study of Morehen et al. (5), the average total EE of 24 female athletes, estimated by doubly labeled water (DLW) method, was 2,693 ± 432 kcal/day, while the EI, assessed by the 4-day food record, was 1,923 ± 232 kcal/day. Several reasons might account for these results: first of all, the underreporting of food records. To this regard, the study of Dasa et al. (3), by comparing EE by DLW to EI by 3-d diet recall, suggested a level of under-reporting of EI of about 22%, supported by the fact that during the observational study no changes in athletes’ BM were measured. When the EI was adjusted by this under reporting, the prevalence of female athletes with low EA decreased (from 34% unadjusted to 5% adjusted), but those of reduced EA did not (57% for both) (47). Another critical point is to ascertain whether these inadequate players’ eating habits are unconscious, possibly due to low nutritional knowledge as suggested by Jenner et al. (48), or conscious decisions, possibly due to social or coaches’ pressures. Finally, other factors favoring insufficient food consumption in athletes could result from excessive daily schedule activities that may not allow proper rest time for eating, or could be a consequence of decrease in appetite post-exercise (49) or, in women, of the menstrual cycle (50).

The second objective of this study was to evaluate the MD adherence of athletes’ diets. Whether MD can be a suitable dietary pattern for competitive athletes is still a matter of debate (51). Undoubtedly, high adherence to MD correlates with lower risk of cardiovascular diseases, metabolic, and neurodegenerative diseases in the general population. The MD may also benefit athletes by preventing excessive ROS production, exercise-induced inflammatory responses, or contributing to injury prevention (51). Our study shows that the majority of soccer players (74%) were adherent to the MD. This result is consistent with other studies that support good adherence of athletes to the MD, even better than that of amateur athletes (52, 53). On the contrary, others have found low MD adherence scores in elite athletes (54, 55). Adherence to the MD should be favored in southern European countries, yet nowadays people are moving away from this traditional dietary pattern (56). To our knowledge, only a few studies have investigated MD adherence in young male football player, finding a medium adherence (57), but no one is available in female football players. Literature data relating to adult female athletes generally show an average-high adherence to the MD, although assessed with different questionnaires (KIDMED-55,58,59- and MEDAS-60). In our volunteers who achieved low MD adherence, the main concerns were related to low frequency of consumption of fruits and vegetables, legumes and fish and higher EI from SFA compared with players with high adherence to MD, although total caloric intake from fat was similar in the two groups.

Some points of strength in this study are worth mentioning: First, studies on female soccer players are limited, and those evaluating nutrient intake and food consumption are often estimated from just one 3-day dietary food record (17, 32, 35). Furthermore, the results of this study help to clarify EE related to female soccer players’ playing positions and their adherence to MD, a dietary regimen widely discussed by sports nutritionists, contributing to support the feasibility of this dietary pattern to ameliorate dietary lipid quality.

Our study also has some limitations, mainly the low number of semi-professional athletes localized in one teams, that limited the power of conclusions, which need to be confirmed in further studies conducted on a larger sample of players. Notwithstanding, studies focusing on food consumption in professional or semi-professional athletes often have similar numbers of participants (57–63). Moreover, athletes self-reported their anthropometric measurements, which may limit the accuracy of these data, body fat percentage were not assessed, RMR and daily physical activity were estimated. The influence of menstrual cycle was not monitored, although its association with nutrient intakes has been recently questioned (64). Finally, the reliability of nutrient intakes assessed by decoding food diaries should be considered with caution, especially for micronutrients, because of the inherent limitations of food composition databases. Even though we recorded food intake, by 3-day food diary two times one month apart, we know that this method might have some disadvantages, such as failing to record foods consumed less frequently or seasonal food (65). In conclusion, the nutritional assessment of the soccer players’ diets highlighted their insufficient EI, due to their low consumption of carbohydrates-rich foods, and the risk of micronutrient deficiency. Good adherence to MD occurred in most of the soccer players, and those with higher adherence showed better dietary lipid quality. Sport nutritionists should pay maximum attention to covering energy needs and in particular to the consumption of foods sources of complex carbohydrates and to optimizing the timing of nutrients intakes, especially proteins, throughout the day. The risk of micronutrient deficiency assessed in the present study, mainly vitamin D, potassium and iodine, is consistent with the eating habits of athletes, who should be encouraged to consume more fatty fish, vegetables and to use iodized salt. Expert nutritional counselling would be highly desirable to correct any dietary mistakes and nutrient timing, ensuring athletes’ expression of their athletic potential and health.

## Data availability statement

The original contributions presented in the study are included in the article/Supplementary material, further inquiries can be directed to the corresponding author.

## Ethics statement

The studies involving humans were approved by the Ethics Committee of the Università degli Studi di Milano. The studies were conducted in accordance with the local legislation and institutional requirements. The participants provided their written informed consent to participate in this study.

## Author contributions

AM: Data curation, Investigation, Writing – original draft. MC: Supervision, Validation, Visualization. DE: Conceptualization, Methodology, Formal analysis, Supervision, Project administration, Writing – original draft.
