# Supplementary material for: Dietary intake and adherence to the Mediterranean diet in semi-professional female soccer players: a cross-sectional study
Source: Front Nutr. 2024 Apr 19;11:1378365. doi: 10.3389/fnut.2024.1378365 (PMC11066240; doi:10.3389/fnut.2024.1378365)
Supplement: Supplementary file 1 [file Table_1.pdf]

Supplementary Material

1 Supplementary Tables

Frequencies of food groups consumption in female soccer players (n=23), according to MEDI-LITE questionnaire.

	portion (g)			
		<1 pz/d	1-1,5 pz/d	>2 pz/d
FRUIT	150	**8,7**	**78,3**	**13,0**
		<1 pz/d	1-2,5 pz/d	>2,5pz/d
VEGETABLES	100	**4,3**	**52,2**	**43,5**
		<1 pz/w	1-2 pz/w	>2/w
LEGUMES	70	**21,7**	**69,6**	**8,7**
		<1 pz/d	1-1,5 pz/d	>1,5/d
CEREALS	130	**0,0**	**82,6**	**17,4**
		<1 pz/w	1-2,5 pz/w	>2,5/w
FISH	100	**26,1**	**60,9**	**13,0**
		<1 pz/d	1-1,5 pz/d	>1,5/d
MEAT AND MEAT PRODUCTS	80	**26,1**	**69,6**	**4,3**
		<1 pz/d	1-1,5 pz/d	>1,5/d
DAIRY PRODUCTS	180	**17,4**	**73,9**	**8,7**
		<1AU/d	1-2AU/d	>2AU/d
ALCOHOL	12	**100**	**0**	**0**
		occasional use	frequent use	regular use
OLIVE OIL		**13,0**	**43,5**	**43,5**

The frequencies with the colored background correspond to the Mediterranean pattern (scored 2). d= daily: w= weekly; pz= portion; AU alcohol unit.
